# FMS-Related Tyrosine Kinase 3 Ligand Promotes Radioresistance in Esophageal Squamous Cell Carcinoma

**DOI:** 10.3389/fphar.2021.659735

**Published:** 2021-05-10

**Authors:** Zuoquan Zhu, Jiahang Song, Junjie Gu, Bing Xu, Xinchen Sun, Shu Zhang

**Affiliations:** ^1^ Department of Radiotherapy, The First Affiliated Hospital of Nanjing Medical University, Nanjing, China; ^2^ Core Facility Center, The First Affiliated Hospital of Nanjing Medical University, Nanjing, China

**Keywords:** fms-related tyrosine kinase 3 ligand, esophageal squamous cell carcinoma, apoptosis, radiotherapy, DNA damage

## Abstract

**Aim:** The FMS-related tyrosine kinase 3 ligand (FL) has an important role in regulating FMS-related tyrosine kinase 3 (Flt-3) activity. Serum FL levels are markedly increased among patients with hematopoietic disease. However, its role in radiation treatment remains unclear. In this study, we investigated the effects of FL on radiotherapy for esophageal squamous cell carcinoma (ESCC).

**Methods:** KYSE150 and KYSE450 cells were stimulated with FL (200 ng/ml). mRNA expression was analyzed using qRT-PCR. Cell viability was checked using CCK-8 assay kits. Proliferation was determined using the EdU assay. Radiosensitivity was detected through a colony-forming assay. Flow cytometry was used to evaluate cell apoptosis. The number of γH2AX foci was verified using an immunofluorescence assay. The change in relative proteins was determined by western blot analysis. The growth of transplanted tumors was demonstrated in nude mice.

**Results:** Our results showed that FL increased the radiation resistance of ESCC cells by promoting clone formation, increasing EdU incorporation, enhancing DNA damage repair, and inhibiting apoptosis. Moreover, the Flt-3 receptor expression significantly increased in ESCC cells after radiation, which may have been an important factor in their radioresistance.

**Conclusion:** Our results suggest that FL increases the radioresistance of esophageal cancer cells and that FL-Flt-3 could be a potential target for enhancing radiosensitivity in ESCC.

## Introduction

Esophageal cancer (EC) is one of the most common cancers worldwide, with the distribution of its histologic subtypes—esophageal squamous cell carcinoma (ESCC) and esophageal adenocarcinoma (EA)—differing depending on geography (Siegel et al., 2020). More specifically, ESCC is a prevalent histological classification in East Asia (Colvin et al., 2017). Radiotherapy (RT) is recommended as an important and effective method for malignant treatment in about half of cancer patients during clinical treatment (Mariette et al., 2015). ESCC patients who have contraindications for surgery or locally advanced disease have a treatment option through RT. However, radioresistance is a major cause of treatment failure, contributing to inadequate cure, relapse, and metastasis (Cohen and Leichman, 2015). Therefore, there is an urgent need to develop possible targets for radiosensitization to improve therapeutic effects on ESCC.

In 1993–1994, the FMS-related tyrosine kinase 3 ligand (FL) was cloned by two independent groups (Lyman et al., 1993; Hannum et al., 1994). FL is a type I transmembrane protein that can be secreted as a soluble dimeric protein. When the FMS-related tyrosine kinase 3 (Flt-3) receptor is activated by membrane-bound or cell-free forms, the growth of progenitor cells is promoted in the bone marrow and blood (Lisovsky et al., 1996a). There is also a strong synergy between FL and other hematopoietic growth factors (Sitnicka et al., 2003). Bone marrow hyperplasia, splenomegaly, lymphadenectasis, and hepatomegaly were evident in mice treated with FL (Tobón et al., 2010). In addition, FL remains at a high level in the majority of leukemias, and mutations in the Flt‐3 gene are frequent genetic lesions in acute myeloid leukemia (Nakao et al., 1996; Stirewalt and Radich, 2003). In contrast, an increased FL plasma concentration can be used as a biomarker for radiation-induced marrow damage and residual hematopoiesis (Bertho et al., 2001; Prat et al., 2006). Moreover, plasma FL levels respond to radiation dose and time of collection (Sproull et al., 2013).

However, little is known about the role of FL in ESCC after RT. In the present study, we investigated the effect of FL on radiation-induced ESCCs and explored the underlying mechanisms.

## Materials and Methods

### Cell Culture and Irradiation Methods

Human ESCC cell lines were obtained from the Shanghai Institute of Cell Biology (Shanghai, China). Cells were cultured in RPMI 1640 medium supplemented with 10% fetal bovine serum at 37°C in a humidified atmosphere with 5% CO_2_. Cells were treated with PBS or recombinant human Flt-3 ligand (0, 5, 10, 20, 40, 80, 160, 320, 200, and 640 ng/ml) (PeproTech, United States), and the cells in the IR group were irradiated with 2, 4, 6, 8, or 10 Gy ionizing radiation X-ray from a medical linear accelerator (Precise accelerator, Elekta, Sweden).

### Quantitative Real-Time PCR

According to the manufacturer’s instructions, mRNA was extracted from cells using Trizol (Invitrogen, Carlsbad, CA, United States) and was reverse transcribed using PrimeScript RT Master Mix (Takara Bio, Otsu, Japan). RT-PCR reactions were performed using a StepOnePlus Real-Time PCR System (Thermo Fisher Scientific, MA, United States) using SYBR Premix Ex Taq II (Takara Bio). The qRT-PCR primers were F: GGT​GGG​AAT​GGG​TCA​GAA​GG (5′-3′) and R: GTA​CAT​GGC​TGG​GGT​GTT​GA (5′-3′) for ACTB, F: AGG​GAC​AGT​GTA​CGA​AGC​TG (5′-3′) and R: GCT​GTG​CTT​AAA​GAC​CCA​GAG (5′-3′) for Flt-3.

### Western Blot Analysis

Total protein was separated from cell lysates using RIPA buffer (Beyotime, Shanghai, China) supplemented with protease and phosphatase inhibitors (Kaygen, Nanjing, Jiangsu Province, China). The protein concentration was quantified using a BCA kit (Beyotime Biotechnology, Shanghai, China). The same amounts of proteins were separated by SDS-PAGE and then transferred to PVDF membranes (BioWorld, Saint Louis, MN, United States). The membranes were blocked with 5% skim milk for 2 h and then incubated overnight with primary antibodies against GAPDH, Flt-3, P-Flt-3, AKT, P-AKT, poly (ADP-ribose), polymerase1 (PARP1), Bad, and P-Bad (Cell Signaling Technology, Boston, MA, United States) at 4°C. On the second day, the membranes were incubated with secondary antibodies (Cell Signaling Technology, Boston, MA, United States) at room temperature for 1 h. Immunoblotted protein signals were detected using an enhanced chemiluminescence detection kit (Thermo Fisher Scientific) on the ChemiDoc XRS imaging system (Quantity One Quantitation software; Bio-Rad Laboratories, Hercules, CA, United States).

### Cell Viability Assay

Cell viability was measured using the CCK-8 assay. The cells were seeded into 96-well plates in triplicates at a density of 5,000 cells per well. After adherence, the cells were treated with or without a single dose of 8 Gy radiation and were stimulated with equal amounts of PBS and FL (200 ng/ml) after 24 h of radiation. At the designated time points after radiation (day 1, day 2, day 3, day 4), CCK-8 solution was added to each well according to the manufacturer’s instructions. The absorbance was measured at a wavelength of 450 nm using a microplate reader (ELx800, BioTek, Winooski, VT, United States). The experiment was repeated thrice.

### Clonogenic Survival Assay

Cells were plated in triplicates into 6-well plates at a density of 1 × 10^2^–10^4^ cells/well and were allowed to adhere for 12 h. Then, the cells were irradiated at doses of 2, 4, 6, and 8 Gy and were cultured in a 5% CO_2_ incubator at 37°C for an additional 10 days. After the end of the assay, cells were stained with Giemsa. Cell survival curves were fitted according to a multi-target single-hit model: S = 1−(1− e^−D/D0^)^N^.

### EdU Assay

The EdU analysis kit (RiboBio, China) was used to assess cell proliferation. ESCC cells were seeded in confocal laser cuvette ESCC plates at a density of 5 × $10^{4}$ cells/well. Next, the cells were incubated in a medium containing 50 μM EdU (C10310-1, RiboBio) for 6 h and processed according to the manufacturer’s instructions. Images were acquired under fluorescence microscopy. The average proportion of EdU-positive cells in three random field of view was analyzed.

### Flow Cytometry Analysis

The cells were seeded in 6-well plates at a density of 1 × 10^5^ cells/well. After 24 h treatment, apoptotic cells were detected using the FITC Annexin V Apoptosis Detection Kit (BD Biosciences, Oxford, United Kingdom). We used flow cytometry to evaluate the rate of apoptosis and repeated all experiments thrice.

### Immunofluorescence Assay

The cells were seeded into a confocal laser cuvette at a concentration of 5 × 10^4^ and were treated with a single dose of 8 Gy. After 24 h of radiation exposure, the cells were exposed to FL (200 ng/ml). The cells were harvested at 0, 2, 8, and 24 h after FL administration. The cells were then fixed in 4% paraformaldehyde for 30 min at room temperature and permeabilized in 0.1% Triton X-100 (Sigma, Santa Clara, CA, United States) for 15 min. The cells were blocked with 5% BSA (Gibco, NY, United States) for 1 h and incubated overnight with the primary antibody γH2AX (1 μg/ml; Abcam, Cambridge, Cambridgeshire, United Kingdom) at 4°C. The cells were washed thrice in TBST every 5 min and then incubated with secondary antibodies (Beyotime Biotechnology) for 1 h. Finally, the cells were treated with 2 μg/ml DAPI (Beyotime Biotechnology) for 5 min and then observed using a confocal fluorescence microscope (Leica, Frankfurt, Germany).

### Xenograft Tumors in Nude Mice

Nude mice (6 weeks old) were obtained from the Nanjing Medical University Animal Center (Nanjing, China). The mice were divided into four groups (n = 5): (a) PBS, (b) FL, (c) IR, and (d) IR + FL. KYSE150 cells were subcutaneously implanted into the right leg of each mouse. Approximately 20 days post-injection, mice in the IR group were exposed to irradiation (8 Gy) once, and 10 μg/d of PBS or FL was administered through intraperitoneal injections. The mice were sacrificed on day 35. Tumor diameters were measured every two days. Tumor volume was calculated according to the following formula: tumor volume (in mm^3^) = (length [L] × width [W]^2^)/2. IHC for P-Flt-3 and Ki 67 (Affinity Biosciences, United States) was also performed.

### Statistical Analyses

The data are expressed as the mean ± standard error of mean of at least three experiments. We used the *t*-test, one-way analysis of variance (ANOVA), and two-way ANOVA to determine the differences between treatment groups. Statistical analyses were performed using GraphPad Prism 5.0 (GraphPad Software, San Diego, CA, United States), and a *p* value of < 0.05 was considered statistically significant.

## Results

### FL Increased Radioresistance in Radiation-Induced ESCC *In Vitro* and *In Vivo*

**FIGURE 1:**
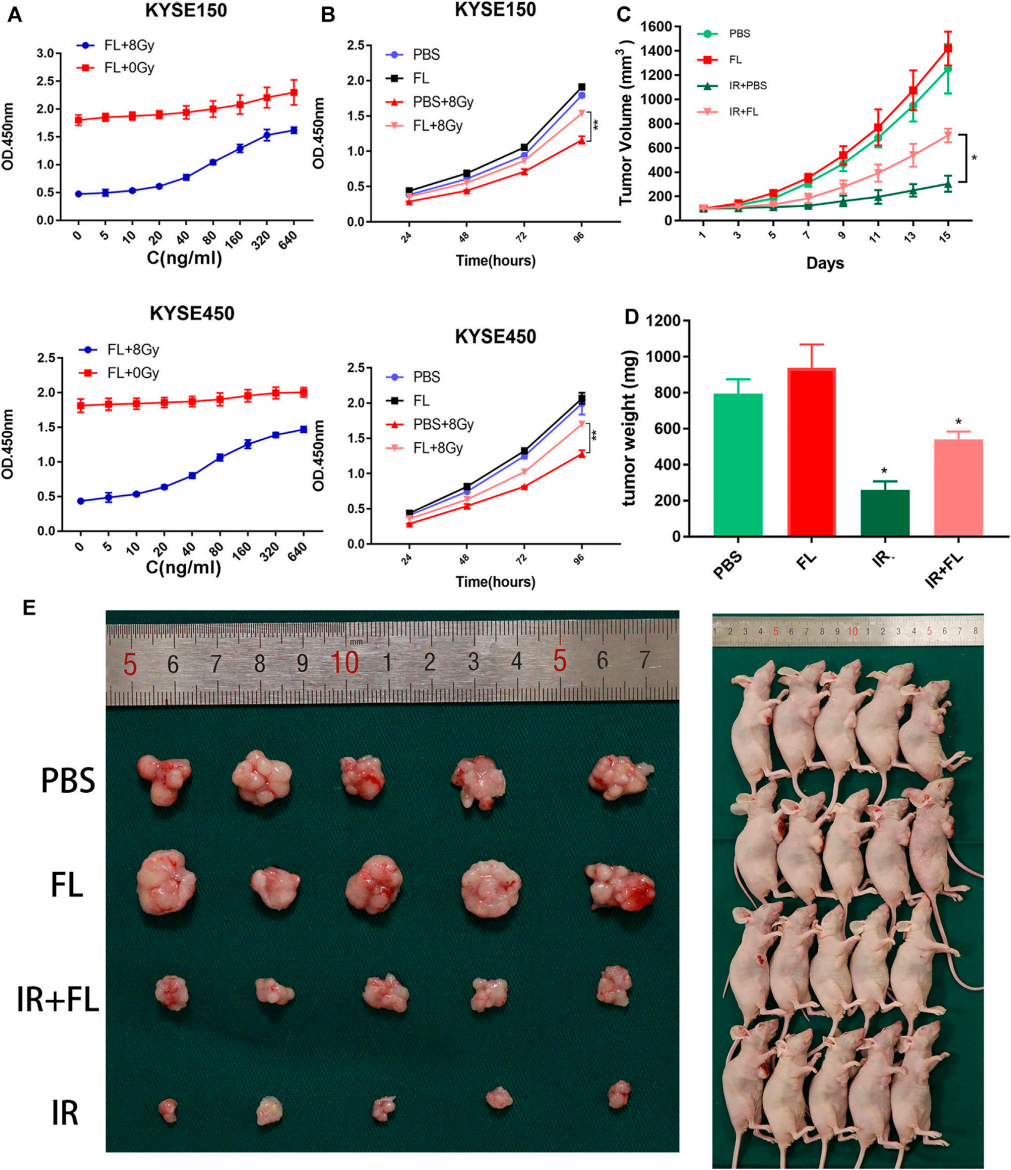
FL promotes viability and tumor growth of ESCCs *in vitro* and *in vivo*. **(A)** The cell viability of ESCC cells in different concentration (0, 5, 10, 20, 40, 80, 160, 320, and 640 ng/ml) of FL (n = 3). **(B)** Cell viability of different time-point after 200 ng/ml FL treatment (n = 3). **(C)** The tumor volume assessed every 2 days (n = 5). **(D)** The tumor weight after sacrificed (n = 5). **(E)** Images of nude mice (n = 5). Mean ± SEM, *p < 0.05, **p < 0 0.01.

KYSE150 and KYSE450 cells were treated with PBS or FL to verify whether FL was related to ESCC tumor growth. Cell viability was detected using a CCK-8 assay. As shown in Figure 1A, the optical density (OD) values significantly increased with the FL concentration (ranging from 5 to 640 ng/ml) in the ionizing radiation X-ray exposure group. Compared to the control, FL (640 ng/ml) increased cell viability up to 342.3 and 338.4% in KYSE150 and KYSE450, respectively. Without radiation, the maximum cell viability induced by FL (640 ng/ml) in KYSE150 and KYSE450 was 127.6 and 110.7%, respectively, compared to the control. In subsequent experiments, 200 ng/ml FL was used. Furthermore, cell viability was detected at 24, 48, 72, and 96 h, and after irradiation, the OD values of the FL group were higher than those of the PBS group (Figure 1B). At 48 h, the cell viability induced in KYSE150 and KYSE450 by FL (200 ng/ml) was 121.6 and 125.5% compared to the control, respectively; at 96 h, these values were 132.5 and 132.7%, respectively. However, there was no significant difference between the non-irradiated groups.

To confirm the effects of FL on ESCC cells *in vivo*, we injected nude mice with KYSE150 cells plus PBS or FL. As shown in Figure 1C, tumor growth significantly slowed in the IR group. The increase in tumor volume was much faster in the FL group than in the PBS group after radiation, and the mean tumor volumes after 15 days were 703 and 305 mm^3^, respectively. By contrast, FL had no significant effect on tumor growth in the non-irradiated groups. Tumor weight was markedly greater in the non-irradiated group, and there was no difference between the FL- and PBS-alone groups. However, the mean tumor weight of the IR plus FL group was larger than that of the IR plus PBS group (540 and 260 g, respectively; Figures 1D,E). Our results indicate that FL weakened the effects of radiotherapy and increased radioresistance in ESCCs.

### FL Promoted Proliferation in ESCC Cell Lines ESCC

**FIGURE 2:**
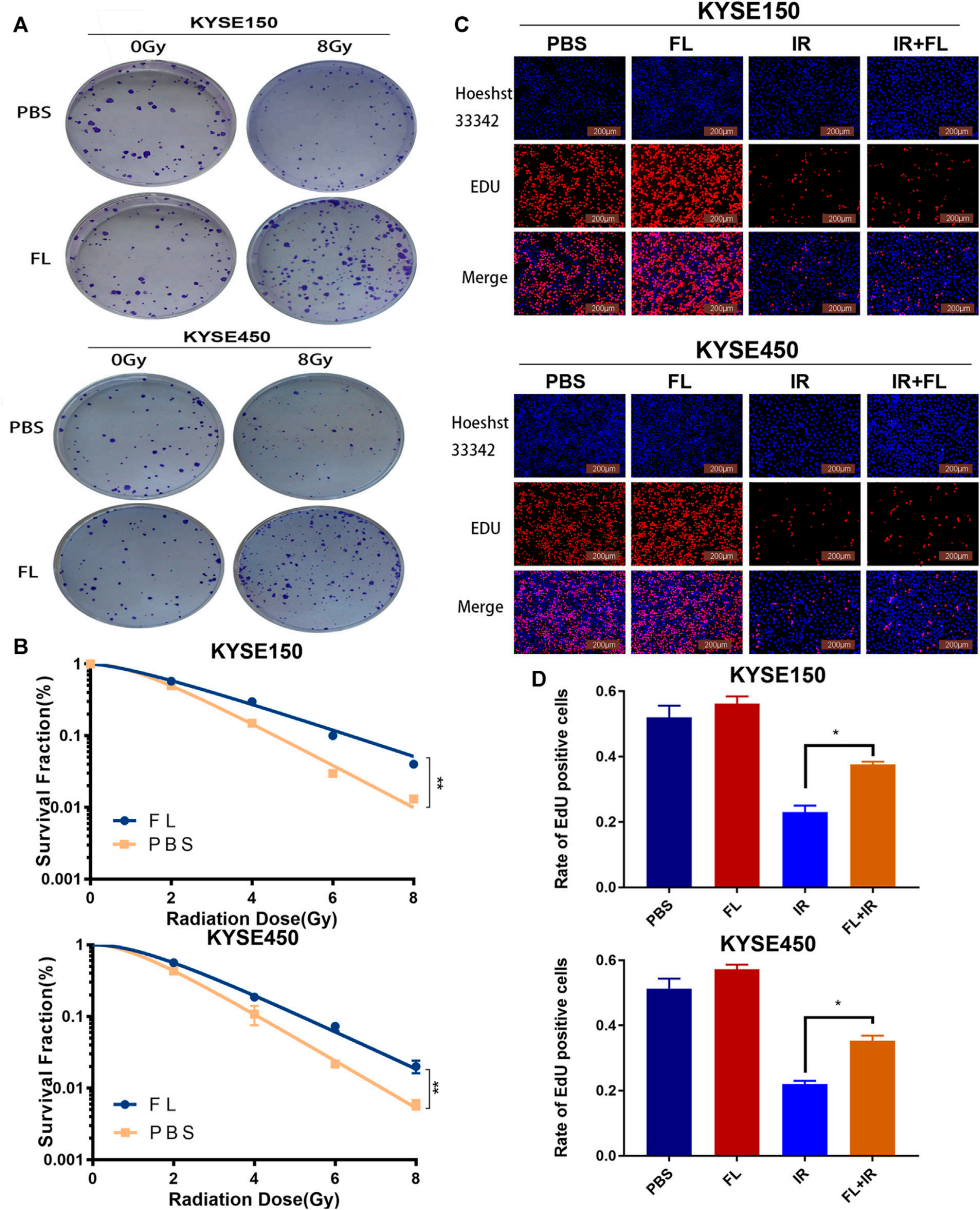
FL promotes radioresistance of ESCC cells *in vitro.* **(A)** The clonogenic survival assays in PBS groups or 200 ng/ml FL administration for 24 h groups after 24 h of 8Gy radiation. **(B)** The survival curves calculated and fitted to a multi-target single-hit model from **(A)**. **(C)** the EdU incorporation assays in ESCC cells (Red fluorescence: EdU-positive cells; Blue fluorescence: total cells). **(D)** Mean rate of EdU positive cells in histogram. Mean ± SEM, N = 3, *p < 0.05, **p < 0.01.

The clonogenic survival assay revealed that ESCC cells in the FL plus radiation group had a higher survival fraction (SF) than those in the PBS plus radiation group (Figures 2A,B). In the FL group, the SF of KYSE150 and KYSE450 at 2 Gy was 0.59 and 0.56, whereas those of the control group was 0.5 and 0.43, respectively. In addition, the mean lethal dose (D_0_) of KYSE150 and KYSE450 in the FL group was 2.34 and 1.66 Gy, compared to the 1.47 and 1.32 Gy in control group, respectively (Supplementary Table S1). Furthermore, we found that FL markedly increased the number of EdU incorporated in the KYSE150 and KYSE450 cells after ionizing radiation. However, there were no obvious changes in the groups without ionizing radiation (Figures 2C,D). So FL has no obvious impact in the groups without ionizing radiation, and it only promoted the proliferation under radiation. These results therefore indicate that FL exerts radiation resistance by promoting the proliferation of ESCC cells.

### FL Enhanced DNA Damage Repair in ESCC Cell Lines ESCC

**FIGURE 3:**
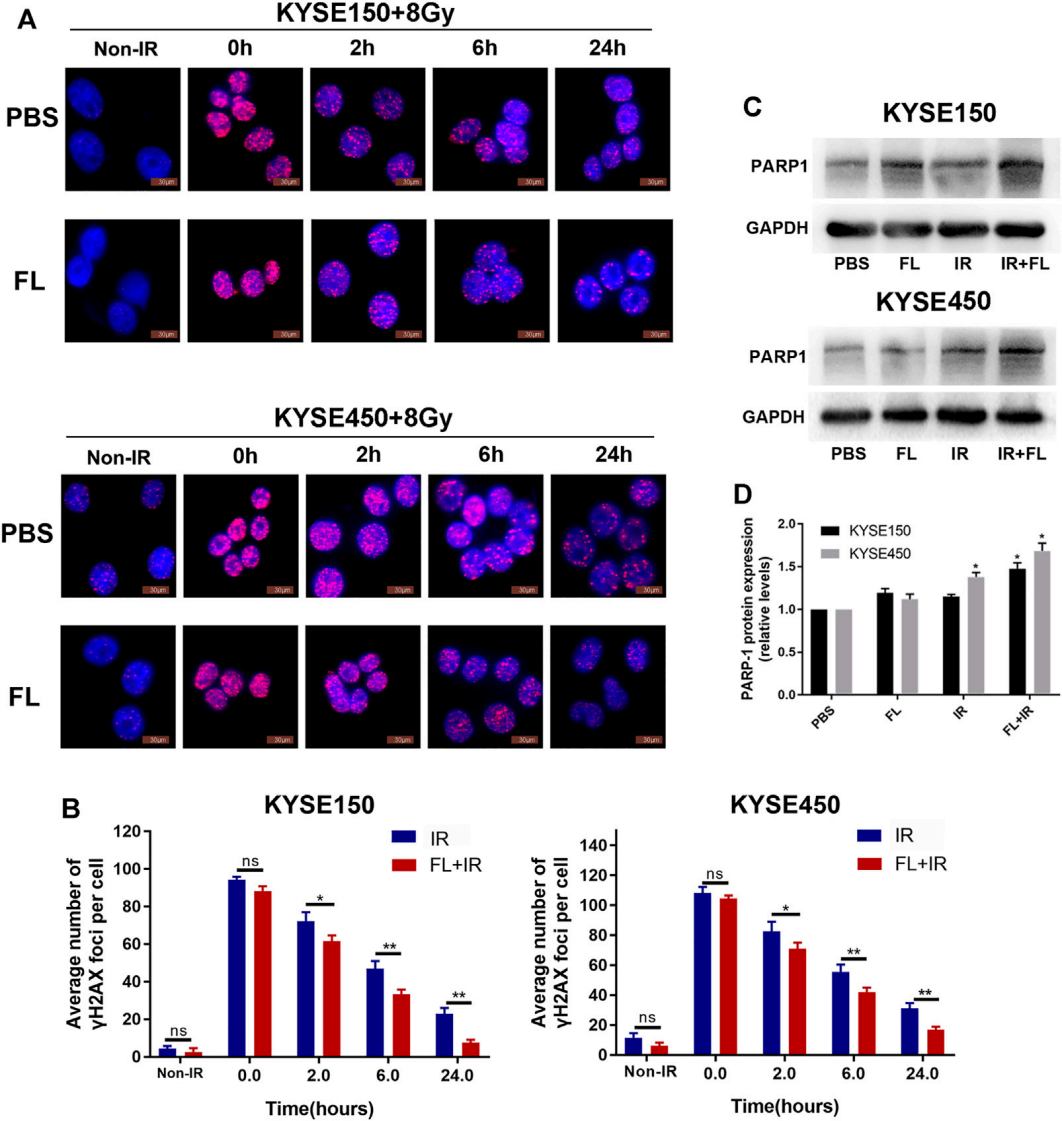
FL reduces IR-induced DNA damage level and increases DNA repair. **(A,B)** Immunofluorescence detection of γH2AX foci in PBS groups or 200 ng/ml FL administration for 24 h groups after 8 Gy radiation different time. Mean ± SEM, N = 3, *p < 0.05, **p < 0 0.01 **(C,D)** PARP1 protein expression in PBS groups or 200 ng/ml FL administration for 24 h groups after 24 h of 8 Gy radiation. GAPDH is an internal reference.

Irradiation can directly cause DNA double-strand breaks; therefore, radiosensitivity depends largely on the ability of tumor cells to repair DNA damage caused by irradiation (Sugase et al., 2017). To assess the effect of FL (200 ng/ml) on 8 Gy IR-induced DNA damage and repair in ESCC cells, we used immunofluorescence to detect the number of gamma-H2AX (γ-H2AX) at different time points (0, 2, 6, and 24 h) after FL administration in the ionizing radiation X-ray exposure group. As shown in Figures 3A,B, the number of *γ*-H2AX in the FL group was not different from that in the PBS group at 0 h. However, the number of *γ*-H2AX in the FL group was lower than that in the PBS group at 2, 6, and 24 h, especially at 24 h. Furthermore, our results showed that the number of descending *γ*-H2AX foci in the FL group was greater than that in the PBS group at the same time point after irradiation. Western blot assays showed that FL (200 ng/ml) increased PARP-1 expression in the 8 Gy radiation group. PARP-1 is a well-known regulator of DNA single-strand break excision repair (Figures 3C,D). These findings suggest that FL enhances DNA damage repair and is involved in the radiation resistance of ESCC cells.

### FL Decreased IR-Induced Apoptosis in ESCC Cell Lines ESCC

**FIGURE 4:**
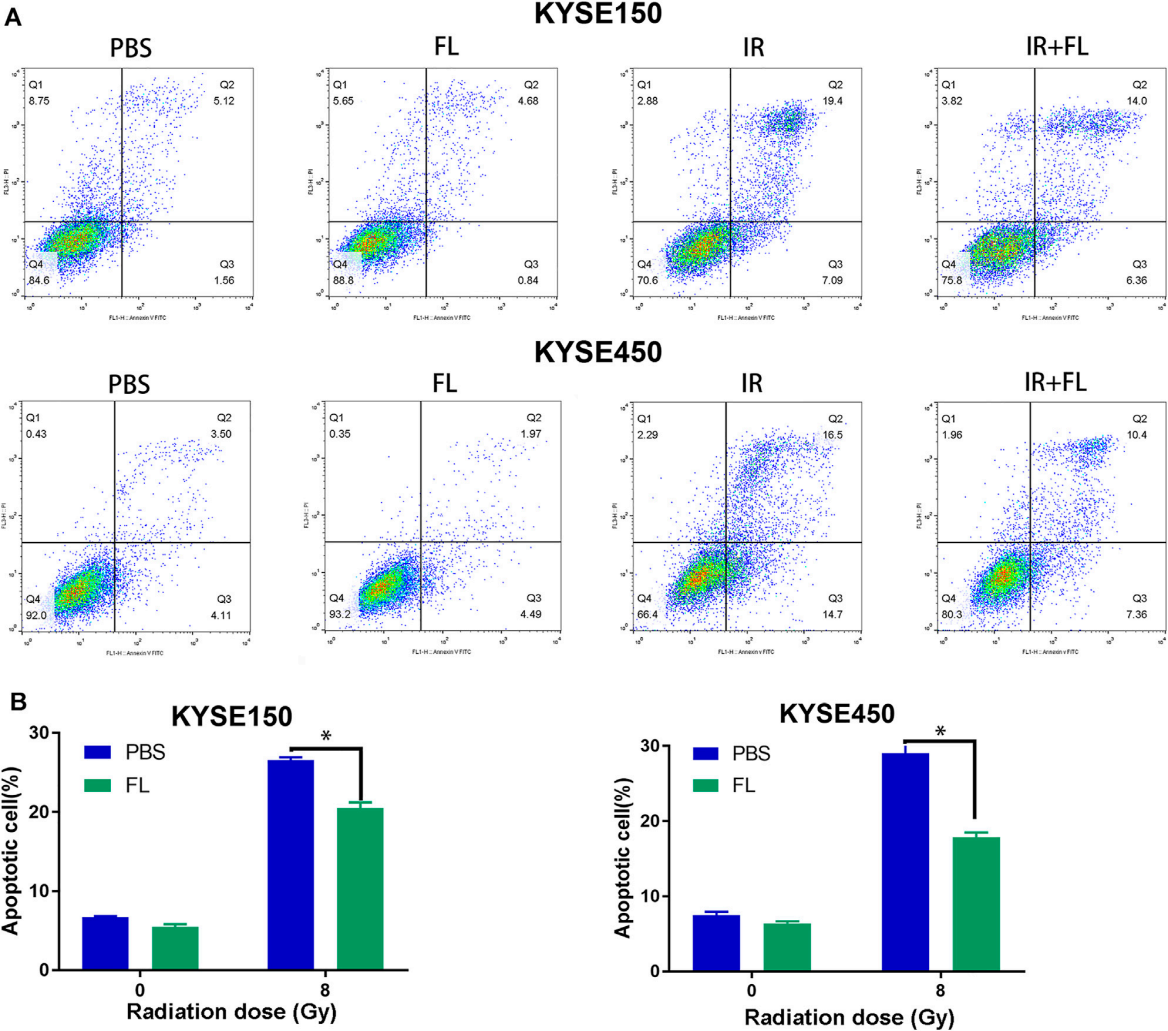
FL decreases IR-induced apoptosis. **(A,B)** Apoptosis ratio in PBS groups or 200 ng/ml FL administration for 24 h groups after 24 h of 8 Gy radiation. Mean ± SEM, N = 3, *p < 0.05.

Apoptosis is an important mechanism that regulates radiosensitivity. Flow cytometry analyses were used to further illustrate the role of FL in IR-induced apoptosis. The total apoptosis rate consisted of the early and late apoptosis populations. As shown in Figures 4A,B, radiation (8 Gy) induced apoptosis in KYSE150 and KYSE450 cells. Furthermore, FL (200 ng/ml) significantly decreased the IR-induced cell apoptosis rate of KYSE150 (from 26.55 to 20.49%) and KYSE450 (from 29.04 to 17.87%). However, without radiation, the apoptosis rate decreased only slightly in KYSE150 (from 6.68 to 5.49%) and KYSE450 (from 7.51 to 6.40%). These results show that FL decreased the IR-induced apoptosis rate to confer radioresistance to ESCC cells.

### Radiation Induced High Expression of the Flt-3 Receptor in ESCC Cell Lines ESCC

**FIGURE 5:**
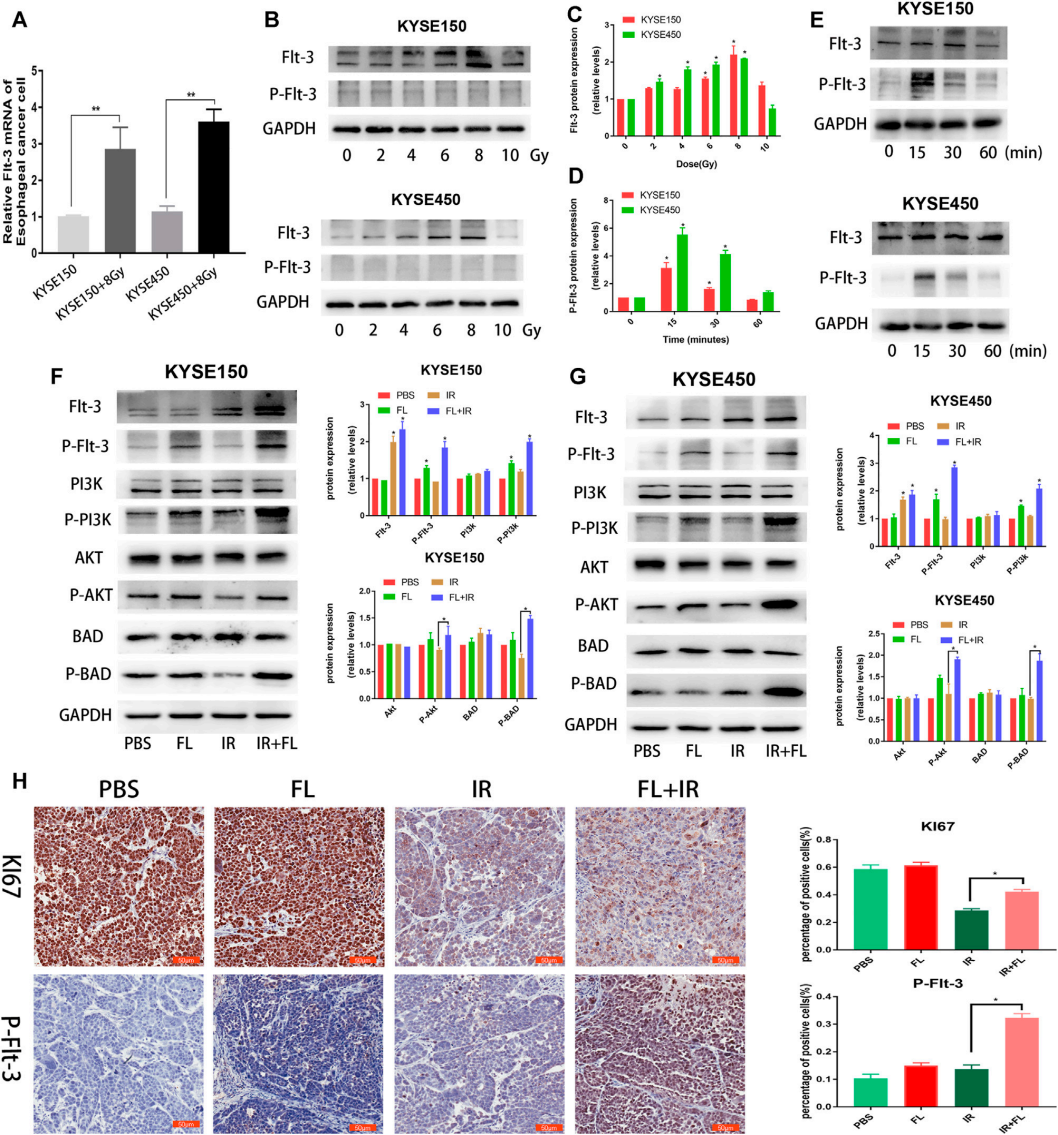
FL induces radioresistance in ESCC cells through Flt-3/AKT/Bad signal pathway. **(A)** mRNA levels of Flt-3 receptor with or without IR in ESCC cells. **(B,C)** Expression and the quantitative analysis results of Flt-3 and P-Flt-3 in ESCC cells after different doses (0, 2, 4, 6, 8, and 10 Gy) radiation. **(D,E)** Expression and the quantitative analysis results of Flt-3 and P-Flt-3 in ESCC cells administrated with 200 ng/ml FL for different time after 24 h of 8 Gy radiation. **(F,G)** The levels and the quantitative analysis results of P-Flt-3/P-PI3K/P-AKT signal proteins and apoptosis-related proteins Bad and P-Bad in PBS groups or 200 ng/ml FL administration for 30 min groups after 24 h of 8 Gy radiation. GAPDH is an internal reference. **(H)** IHC images of nude mice and IHC analysis of P-Flt-3 and Ki 67 in tumor sections of KYSE150 bearing nude mice. Data show mean ± SEM *p < 0.05.

FL can activate the Flt-3 receptor via membrane-bound forms; hence, we detected the mRNA and protein levels of the Flt-3 receptor in ESCC cells. As shown in Figure 5A, irradiation (8 Gy) upregulated the expression of Flt-3 receptor mRNA in KYSE150 and KYSE450 cells. Furthermore, we found that this irradiation-induced upregulation of the Flt-3 receptor is dose-dependent (0, 2, 4, 6, and 8 Gy), whereas the Flt-3 receptor phosphorylation did not significantly change in either KYSE150 or KYSE450 cells 24 h after irradiation (Figures 5B,C). Furthermore, the time course experiments showed that 200 ng/ml FL led to a rapid and transient phosphorylation of Flt-3, with the peak level occurring at 15 min (Figures 5D,E); however, the Flt-3 receptor level did not change within 1 h after FL administration. These results suggest that IR-induced increased expression of the Flt-3 receptor may play a critical role in the FL-induced radioresistance of ESCC cells.

### FL Intensified IR-Induced Flt-3/PI3K/AKT Pathway Activation

Downstream-related proteins were analyzed further and we detected the Flt-3/PI3K/AKT signaling pathway and apoptosis-related proteins Bad (pro-apoptotic protein) and P-Bad (anti-apoptotic protein). As shown in Figures 5F,G, the phosphorylation of Flt-3, PI3K, and AKT significantly increased when FL was combined with IR, compared with FL alone. P-Bad (ser136) protein levels were upregulated in the FL plus IR group, compared to the FL alone group. Furthermore, IHC analysis of the xenograft nude mice tumor tissues showed that P-Flt-3 receptor expression significantly increased in the FL plus IR group compared to the control, FL, and IR alone groups. Moreover, Ki 67 expression in the FL plus IR group was significantly higher than that in the FL alone group (Figure 5H). These results indicate that IR-induced upregulation of Flt-3 receptor and FL expression further activated the receptor and its downstream signaling pathway, which resulted in radioresistance in ESCC cells.

## Discussion

Cytokines are increased in hematologic malignancies and FL has a vital role in the generation of mature peripheral leukocytes (McKenna et al., 2000), including the common lymphoid progenitor (Sitnicka et al., 2002). FL, a potent hematopoietic cytokine, mobilizes hematopoietic cells into the blood and lymphoid organs, and promotes the growth and differentiation of progenitor and stem cells both *in vivo* and *in vitro* (Antonysamy and Thomson, 2000). In addition to its effects to the hematopoietic progenitor and stem cells, FL also increases the number of dendritic cells in the peripheral blood and is involved in maintaining the viability of irradiated hematopoietic stem cells (MacVittie and Farese, 2002). In the current study, we found that FL increased the viability of KYSE150 and KYSE450 cells in a dose-dependent manner after radiation exposure. However, FL treatment did not affect cell viability in the non-irradiated group. By contrast, FL promoted tumor growth in the irradiation exposure group; nevertheless, there was no significant difference between this and the no irradiation exposure group *in vivo*. These results indicate that FL weakened the effects of radiotherapy on ESCC.

Furthermore, we explored the reasons behind FL’s attenuation of radiotherapy effects. The clonogenic survival assay and the EdU incorporation assay were also employed for a more sensitive evaluation of proliferation after radiation (Feng et al., 2010; Li et al., 2017). We found that FL promoted colony formation and increased the number of EdU incorporated into KYSE150 and KYSE450 cells. On the other hand, previous studies have shown that FL expands the number of functionally competent human DCs *in vivo* (*Maraskovsky et al., 2000*). Ultimately, our results suggest that FL’s promotion of cell proliferation ability plays a role in the radioresistance of ESCC cells.

We also found that FL reduced DNA damage, enhanced DNA damage repair, and further reduced radiation-induced apoptosis in KYSE150 and KYSE450 cells. DNA damage repair plays a significant role in radiosensitivity after radiation (Qian et al., 2014). The phosphorylated form of H2AX (γH2AX) is used as a marker of DNA double-strand breaks (Sak and Stuschke, 2010). Poly (ADP-ribose) polymerase-1 (PARP-1) also participates in the modulation of cellular responses to DNA damage (Smith, 2001). Similarly, Veuger et al. revealed that the PARP-1 protein mediates the repair of both single- and double-strand DNA breaks after radiation (Veuger et al., 2003). Furthermore, evidence indicates that the apoptotic pathway is an essential mechanism for the regulation of radiosensitivity (Raleigh and Haas-Kogan, 2013). These results show that FL increased the radiation resistance of ESCC cells by promoting clone formation, increasing EdU incorporation, enhancing DNA damage repair, and inhibiting apoptosis in ESCCs.

FL can activate the Flt-3 receptor via membrane-bound and soluble forms to mobilize progenitor cells of the bone marrow (Tobón et al., 2010). In this study, we demonstrated for the first time that the expression of the Flt-3 receptor is significantly higher in ESCC cells after radiation. We inferred that IR-induced increased expression of the Flt-3 receptor is an important reason for the radioresistance of ESCC cells.

**FIGURE 6:**
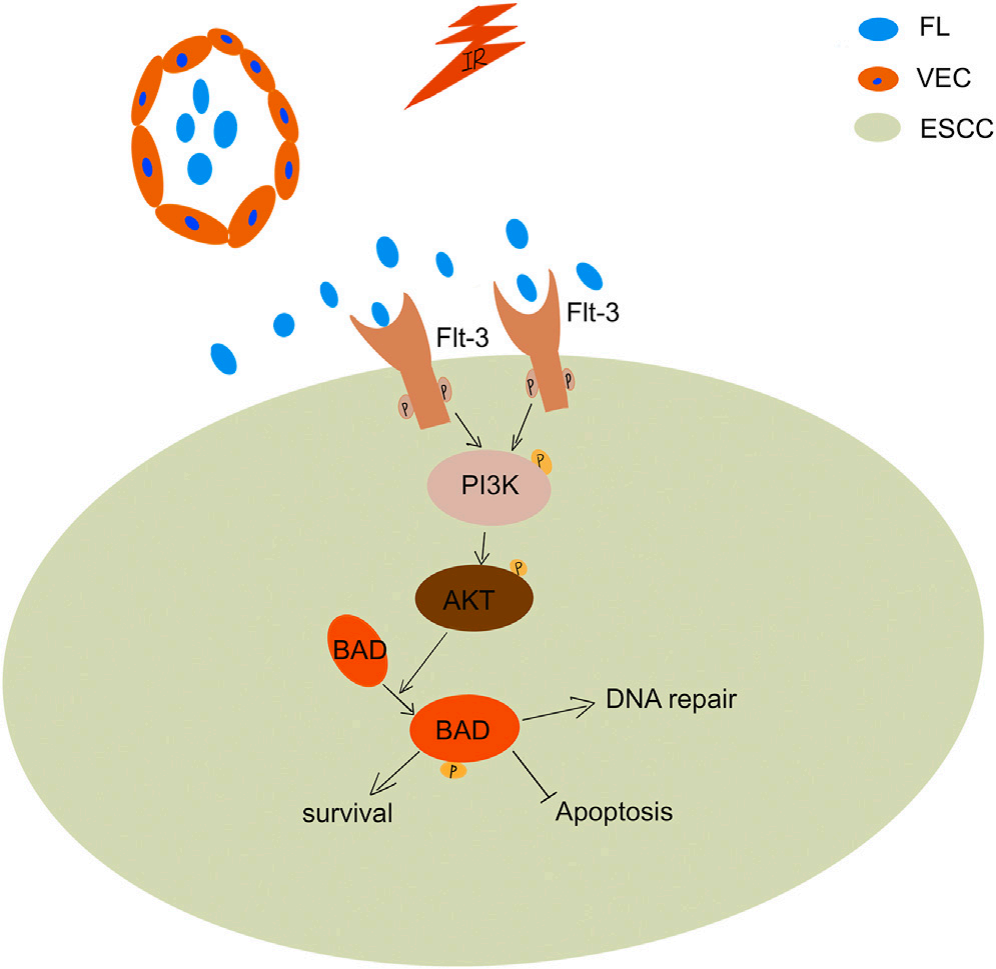
Relevant mechanism of FL mediated the radioresistance of ESCCs during the radiotherapy. Radiation significantly increase Flt-3 receptor expression in ESCCs. FL phosphorylate the increased Flt-3 receptor, which further activate the PI3K/Akt pathway leading to BAD phosphorylation.

Previous studies have shown that FL induces phosphorylation of Flt-3 receptor signaling and enhances cell proliferation (Lisovsky et al., 1996b). Our study found that FL promoted the phosphorylation of the Flt-3 receptor after radiation to inhibit IR-induced apoptosis. Phosphorylation of the Flt-3 receptor reportedly activates PI3K and downstream AKT, and subsequently, the phosphorylation of BAD protein at Ser-136, leading to anti-apoptotic effects (Kim et al., 2006). Similar experimental results were obtained in our study. The BAD protein phosphorylation changes its binding ability to Bcl-2, resulting in a low ability to heterodimerize with relative survival proteins (Zha et al., 1996). In our study, radiation significantly increased Flt-3 receptor expression, which was phosphorylated by FL in ESCC cells. This phosphorylation of the Flt-3 receptor further activated PI3K/AKT/BAD signaling pathways and promoted clone formation, increased EdU incorporation, enhanced DNA damage repair, and inhibited apoptosis in IR-induced ESCC (Figure 6).

In conclusion, our results showed that FL boosted the radioresistance of ESCC cells both *in vitro* and *in vivo*. This radioresistance involves improving vitality, weakening DNA damage, enhancing DNA repair, and inducing anti-apoptosis. Moreover, Flt-3 receptor expression significantly increased in ESCC cells after radiation, which may be an important reason for the radioresistance. Therefore, FL-Flt-3 is a potential molecular target for enhancing radiosensitivity in ESCC.

## Manuscript Contribution to the Field

In this manuscript, we found that the Fms-related tyrosine kinase 3 ligand (FL) boosted the radioresistance of ESCCs both *in vitro* and *in vivo*. The radioresistance of ESCCs involve in improving vitality, weakening DNA damage, enhancing DNA repair and inducing anti-apoptosis. Moreover, Flt-3 receptor expression increased significantly in ESCC cells after radiation, which is an important reason for the radioresistance of esophageal squamous cell carcinoma This is the first report that irradiation can promote the expression of Flt-3 receptor on tumor cells. We hypothesized that high expression of Flt-3 receptor was phosphorylated by FL. Phosphorylation of Flt-3 receptor further activated PI3K/AKT/BAD signal pathways and led to promote clone formation, increase EdU incorporation, enhance DNA damage repair and inhibit apoptosis in IR-induced ESCCs. Therefore, FL - Flt-3 is a potential molecular target for enhancing radiosensitivity of esophageal squamous cell carcinoma.

## Data Availability

The raw data supporting the conclusions of this article will be made available by the authors, without undue reservation.
